# Distinct effects of current suicidal symptoms and lifetime suicide attempts on C-reactive protein levels and executive function in young people with major affective disorders

**DOI:** 10.1007/s00406-026-02293-z

**Published:** 2026-06-22

**Authors:** Ju-Wei Hsu, Li-Chi Chen, Ya-Mei Bai, Shih-Jen Tsai, Mu-Hong Chen

**Affiliations:** 1 Department of Psychiatry, Taipei Veterans General Hospital, No. 201, Shih-Pai Road, Sec. 2, Taipei, 11217 Taiwan; 2 Department of Psychiatry, College of Medicine, National Yang Ming Chiao Tung University, Taipei, Taiwan; 3 Department of Psychiatry, General Cheng Hsin Hospital, Taipei, Taiwan

**Keywords:** Current suicidal symptoms, Suicide attempt history, Young people, Major affective disorders, C-reactive protein, Executive dysfunction

## Abstract

**Background:**

This study addresses the complex associations between current suicidal symptoms, lifetime suicide attempt history, C-reactive protein (CRP) levels, and executive dysfunction among young people with major affective disorders.

**Methods:**

A total of 171 young people with major affective disorders presenting varying levels of suicidal symptoms and 97 healthy young people aged 12 to 24 years were recruited for this study. Current suicidal symptom severity was classified as none, mild, or strong if an individual has scores of 0, 2–3, and ≥ 4, respectively, on item 10 of the Montgomery–Åsberg Depression Rating Scale (MADRS). The presence of a lifetime history of suicide attempts was also determined. All participants had CRP levels measured and underwent the Wisconsin Card Sorting Test (WCST).

**Results:**

Generalized linear models (GLM) with adjustments for demographic characteristics, diagnoses, and non-suicidal depressive symptoms indicated that patients presenting strong suicidal symptoms had the highest CRP levels (*p* = 0.004) and percentage of nonperseverative errors on the WCST (*p* = 0.002), whereas those with mild suicidal symptoms were more likely to have intermediate CRP levels relative to non-suicidal young people. Only young people with a history of suicide attempts exhibited an increased percentage of perseverative errors on the WCST (*p* = 0.023) compared with the healthy controls.

**Discussion:**

Our findings suggest that CRP levels and the percentage of nonperseverative errors on the WCST served as concurrent markers of suicidality, whereas the percentage of perseverative errors served as a trait marker of suicidality among young people with major affective disorders.

## Introduction

In Taiwan, suicide has emerged as the second leading cause of death among adolescents and young adults aged 15 to 24 years. A concerning uptick in the suicide rate for this demographic has been noted, more than doubling in a span of two decades from 4.0 per 100,000 individuals in 2000 to 10.7 per 100,000 individuals in 2022 [1]. Annual adolescent and young adult suicide rates climbed 11.5% between 2014 and 2019 in Taiwan [1, 2], mirroring similar trends in the United States [3]. The World Health Organization Mortality Database indicates that the highest age-standardized suicide rate in the United States was 15.5 per 100,000 among men and boys [3]. Additionally, from 2009 to 2020, young male suicide rates increased by 3.8% annually, whereas young female suicide rates increased by 6.7% from 2007 to 2017 [3].

Increasing evidence has indicated C-reactive protein (CRP) to be a potential biomarker of suicidality, including suicidal ideation, suicide attempts, and even suicide death. A meta-analysis of 3387 patients with depression, including 1269 with suicidal symptoms, revealed that patients with suicidal symptoms exhibited higher CRP levels (standardized mean difference = 0.80) than those without such symptoms [4]. Specifically, Aguglia et al. measured CRP levels among patients with differing suicide attempt histories and demonstrated that a cut-off point of CRP > 4.65 mg/L predicted high-lethality suicide attempts, with a sensitivity of 0.68 and a specificity of 0.71 [5]. A cross-sectional study of 600 inpatients with depression (including 520 patients with a lifetime history of suicide attempts and 80 without) found that the risk of suicide increased with higher CRP levels in a dose-response fashion [6]. Furthermore, compared with non-suicidal individuals, those with strong suicidal ideation or a history of suicide attempts had greater CRP levels [7]. However, previous studies have typically assessed CRP levels among mid-life patients with depression (mean age = approximately 45 years), but not among adolescents and young adults [4–7]. Findings from a small sample of 37 young people with depression (aged 10 to 25 years) with suicidal symptoms and 39 healthy controls revealed no between-group difference in CRP levels [8]. Conversely, Priya et al. examined the CRP levels of 42 young adults with a history of suicide attempts and 42 healthy controls and found a positive relationship between CRP levels and suicide risk [9]. These inconsistent findings call for further investigation.

Executive dysfunction may also play a key role in suicide pathophysiology [10, 11]. Le et al. have suggested that executive dysfunction is an endophenotype of suicidality, regardless of diagnosis (schizophrenia, bipolar disorder, or major depressive disorder) [10]. A 1-year follow-up study of 104 adults with major depressive disorder indicated that executive dysfunction at baseline was associated with subsequent suicidal ideation (odds ratio = 5.32) and suicide attempts (odds ratio = 8.81) [12]. Chen et al. assessed executive function among 77 adolescents with depression (including 52 with a prior history of suicide attempts and 25 without) using the Wisconsin sorting card test (WCST) and found that those with a history of suicide attempts performed significantly worse in terms of executive function than those without [13]. By contrast, Itzhaky et al. found that among a cohort of 100 psychiatric inpatient adolescents, those with a mood disorder and history of suicide attempts performed better on the WCST than those with a mood disorder and no such history [14]. Ortuño-Sierra et al. found no association between executive function and suicidal symptoms in a sample of 83 adolescents at risk for suicide and 83 healthy controls [15]. Further studies are required to clarify these inconsistent findings.

Our study investigated the effects of current suicidal symptom severity (a concurrent marker of suicidality) and lifetime suicide attempts (a trait marker) on CRP levels and executive function among young people with major affective disorders. We hypothesized that both current suicidal symptoms and lifetime suicide attempts would be associated with increased CRP levels and higher executive function deficits among young people with major affective disorders.

## Methods

### Participants

 In the current study, we recruited young people aged 12 to 24 years diagnosed with major affective disorders, specifically bipolar disorder and major depressive disorder. Participants were required to have no prior history of significant physical illnesses, such as epilepsy, autoimmune disorders, or neurovascular diseases, and no lifetime history of intellectual disability, schizophrenia, organic mental disorders, or alcohol and substance use disorders. The Montgomery–Åsberg Depression Rating Scale (MADRS) was utilized to assess the overall depressive symptoms [16]. Using the scores from item 10 of the MADRS, which assesses suicidal symptoms, participants were categorized into three groups based on cut-off points of 2 and 4: 0 (enjoys life or takes it as it comes) (non-suicidal group), 2 (weary of life; only fleeting suicidal thoughts) or 3 (mild suicidal symptom group), and ≥ 4 (4: feels better off dead; suicidal thoughts common and considered as possible solution but no specific plans/intention; 6: explicit plans for suicide when there is an opportunity or active preparation for suicide; 5: between 4 and 6) (strong suicidal symptom group) [16, 17]. Prior clinical research protocols commonly treated MADRS item 10 values ≥ 4 as indicators of strong suicide risk [18, 19]. Participants who scored 1 on item 10 of the MADRS were excluded from the current study due to the ambiguity of suicidal symptoms. We also recruited healthy controls who had no history of the aforementioned physical conditions or psychiatric disorders. In the present study, the Structured Clinical Interview for DSM-5 Disorders (SCID) was performed to ensure a diagnosis in the case group and no mental disorder diagnosis in the healthy control group. In addition, lifetime history of suicide attempts was assessed using the SCID. Corresponding author Dr. MHC performed the SCID and rated the clinical scales. The lifetime history of the significant physical illnesses was acquired based on the self- and parent-reported health questionnaires designed in the present study. All procedures contributing to this study adhered to the ethical standards set forth by the relevant national and institutional committees on human experimentation, in accordance with the Helsinki Declaration of 1975, as revised in 2008. All protocols involving human subjects or patients received approval from the Institutional Review Boards of Taipei Veterans General Hospital. Informed consent was obtained in writing from all participants, as well as from the parents of adolescent subjects.

### Assessment of CRP levels

 Using enzyme-linked immunosorbent assay (ELISA) kits from R&D Systems (Minneapolis, MN, USA), we measured the CRP levels in each participant. Fasting serum samples were collected in serum separator tubes (SSTs) and allowed to clot for 30 min between 9:00 AM and 12:00 PM. Subsequently, all samples were stored at -80 °C until analysis. Assays were conducted following the manufacturer’s instructions. The final absorbance of the mixture was measured at 450 nm using an ELISA plate reader (Bio-Tek Power Wave Xs) and analyzed with Bio-Tek’s KC Junior software (Winooski, VT, USA). The standard range was determined according to the manufacturer’s guidelines, and a linear regression R² value of ≥ 0.95 was indicative of a reliable standard curve.

### Assessment of executive function

 The WCST was administered in this study to evaluate executive function. Successful performance on the WCST necessitates strategic planning, organized search strategies, the ability to utilize environmental feedback to adjust cognitive sets, goal-directed behavior, and the regulation of impulsive responses. The WCST has been employed extensively in our prior research [20, 21].

### Statistical analysis

 For between-group comparisons, the F-test was used for continuous variables and Pearson’s test was used for categorical variables. We conducted generalized linear models (GLMs), adjusting for demographic factors (age, sex, body mass index [BMI], and years of education), diagnoses, and nonsuicidal depressive symptoms reflected by subtotal scores from MADRS items 1 − 9, to evaluate the main effects of current suicidal severity conditions (as indicated by MADRS item 10 scores categorized as 0 vs. 2 or 3 vs. ≥ 4) and lifetime suicide attempts, as well as the interactive effect of these two conditions on executive function. Following these adjustments, we also conducted additional GLMs with a gamma log link to evaluate the main effects of current suicidal severity conditions and lifetime suicide attempts, as well as their interactive effect, on CRP levels. We further focused on the statistically significant effects of the GLMs for the further post-hoc analyses. A two-tailed P value of less than 0.05 was considered statistically significant. All data processing and statistical analyses were performed using the SPSS version 17 software (SPSS Inc., Chicago, IL).

### Data availability

The datasets collected and/or analyzed during the current study are available from the corresponding author upon reasonable request. However, they are not publicly accessible due to ethical regulations governing clinical trials in Taiwan.

## Results

In this study, we included a total of 171 young people diagnosed with major affective disorders and 97 healthy controls. Among the participants, 164 exhibited no current suicidal symptoms, while 80 demonstrated mild suicidal symptoms (as indicated by a score of 2 or 3 on item 10 of the MADRS). Additionally, 24 participants scored ≥ 4 at the MADRS item 10. Furthermore, 120 participants had no prior history of suicide attempts, whereas 51 had a documented history of attempted suicide (Tables 1 and 2). Before the adjustment of covariates, Table 1 demonstrated that patients with strong suicidal symptoms exhibited the worst executive function measured by the percentage of nonperseverative errors on the WCST (*p* = 0.003) and the highest CRP levels (*p* = 0.001) compared with the other two groups. In addition, patients with a prior history of suicide attempts had the highest percentage of nonperseverative errors (*p* = 0.001) and perseverative errors on the WCST (*p* = 0.005), as well as CRP levels (*p* = 0.003) (Table 2).

**Table 1:** Demographic and clinical characteristics of youth with varying levels of concurrent suicidal symptoms

	Severity of suicidal symptoms based the MADRS item 10 scores (*n* = 268)				
	0 (A) (*n* = 164)	2 or 3 (B) (*n* = 80)	≥ 4 (C) (*n* = 24)	*p*-value	Post-hoc
Age (years, SD, n, %)	18.05 (3.76)	18.04 (3.10)	17.42 (3.20)	0.704	
Sex (n, %)				0.009	
Male	62 (37.8)	17 (21.3)	4 (16.7)		
Female	102 (62.2)	63 (78.7)	20 (83.3)		
BMI (SD)	21.51 (3.87)	22.29 (4.52)	23.68 (6.21)	0.049	C > A
Education years (years, SD)	11.59 (3.33)	11.50 (2.57)	11.08 (2.67)	0.755	
Diagnosis (n, %)				0.001	
Bipolar disorder	34 (20.7)	34 (42.5)	8 (33.3)		
Euthymic state	18 (11.0)	0 (0.0)	0 (0.0)		
Depressive state	16 (9.7)	34 (42.5)	8 (33.3)		
Major depressive disorder	33 (20.1)	46 (57.5)	16 (66.7)		
Euthymic state	9 (5.5)	0 (0.0)	0 (0.0)		
Depressive state	24 (14.6)	46 (57.5)	16 (66.7)		
Control group (n, %)	97 (59.1)				
Subtotal MADRS items 1–9 scores (SD)	5.78 (8.02)	22.48 (5.09)	29.96 (5.94)	< 0.001	C > B> A
History of attempted suicide (n, %)	15 (9.1)	25 (31.3)	12 (50.0)	< 0.001	C > B> A
WCST (SD)					
Number of categories completed	5.71 (1.04)	5.65 (1.46)	5.25 (1.39)	0.219	
% Nonperseverative errors	9.16 (4.90)	10.36 (6.76)	13.50 (6.22)	0.003	C > B~A
% Perseverative errors	10.98 (8.75)	11.20 (7.14)	13.00 (6.39)	0.524	
CRP (ng/ml, SD)	878.40 (1479.19)	1370.24 (2003.31)	2614.00 (4762.13)	0.001	C > B~A

*MADRS* Montgomery–Åsberg depression rating scale, *SD* standard deviation, *BMI* body mass index, *WCST* Wisconsin card sorting test, *CRP* C-reactive protein

**Table 2:** Demographic and clinical characteristics of youth with and without lifetime history of attempted suicide

		Patients with major affective disorders (*n* = 171)			
	Control group (*n* = 97)	Without lifetime SA (B) (*n* = 120)	With lifetime SA (C) (*n* = 51)	*p*-value	Post-hoc
Age (years, SD, n, %)	16.22 (3.41)	19.06 (3.24)	18.86 (3.05)	< 0.001	C ~ B> A
Sex (n, %)				0.001	
Male	44 (45.4)	29 (24.2)	10 (19.6)		
Female	53 (54.6)	91 (75.8)	41 (80.4)		
BMI (SD)	21.05 (3.65)	21.95 (4.07)	23.59 (5.62)	0.003	C ~ B> A
Education years (years, SD)	10.04 (3.09)	12.43 (2.85)	12.16 (2.39)	< 0.001	C ~ B> A
Diagnosis (n, %)				< 0.001	
Bipolar disorder		44 (36.7)	32 (62.7)		
Euthymic state		11 (9.2)	7 (13.7)		
Depressive state		33 (27.5)	25 (49.0)		
Major depressive disorder		76 (63.3)	19 (37.3)		
Euthymic state		9 (7.5)	0 (0.0)		
Depressive state		67 (55.8)	19 (37.3)		
Subtotal MADRS items 1–9 scores (SD)	0.61 (1.08)	19.68 (8.66)	20.47 (8.78)	< 0.001	C ~ B> A
MADRS item 10 scores				< 0.001	
0	97 (100.0)	53 (44.2)	14 (27.5)		
2 or 3	–	55 (45.8)	25 (49.0)		
≥ 4	–	12 (10.0)	12 (23.5)		
WCST (SD)					
Number of categories completed	5.87 (0.69)	5.55 (1.22)	5.49 (1.83)	0.093	
% Nonperseverative errors	9.01 (5.02)	9.59 (5.62)	13.11 (6.81)	0.001	C > B~A
% Perseverative errors	9.87 (7.25)	11.00 (6.76)	14.35 (11.28)	0.005	C > B~A
CRP (ng/ml, SD)	743.30 (1164.25)	1179.42 (1829.38)	2055.64 (3766.10)	0.003	C > B~A

*SA* suicide attempt, *MADRS* Montgomery–Åsberg depression rating scale, *SD* standard deviation, *BMI* body mass index, *WCST* Wisconsin card sorting test, *CRP* C-reactive protein

**Fig. 1:**
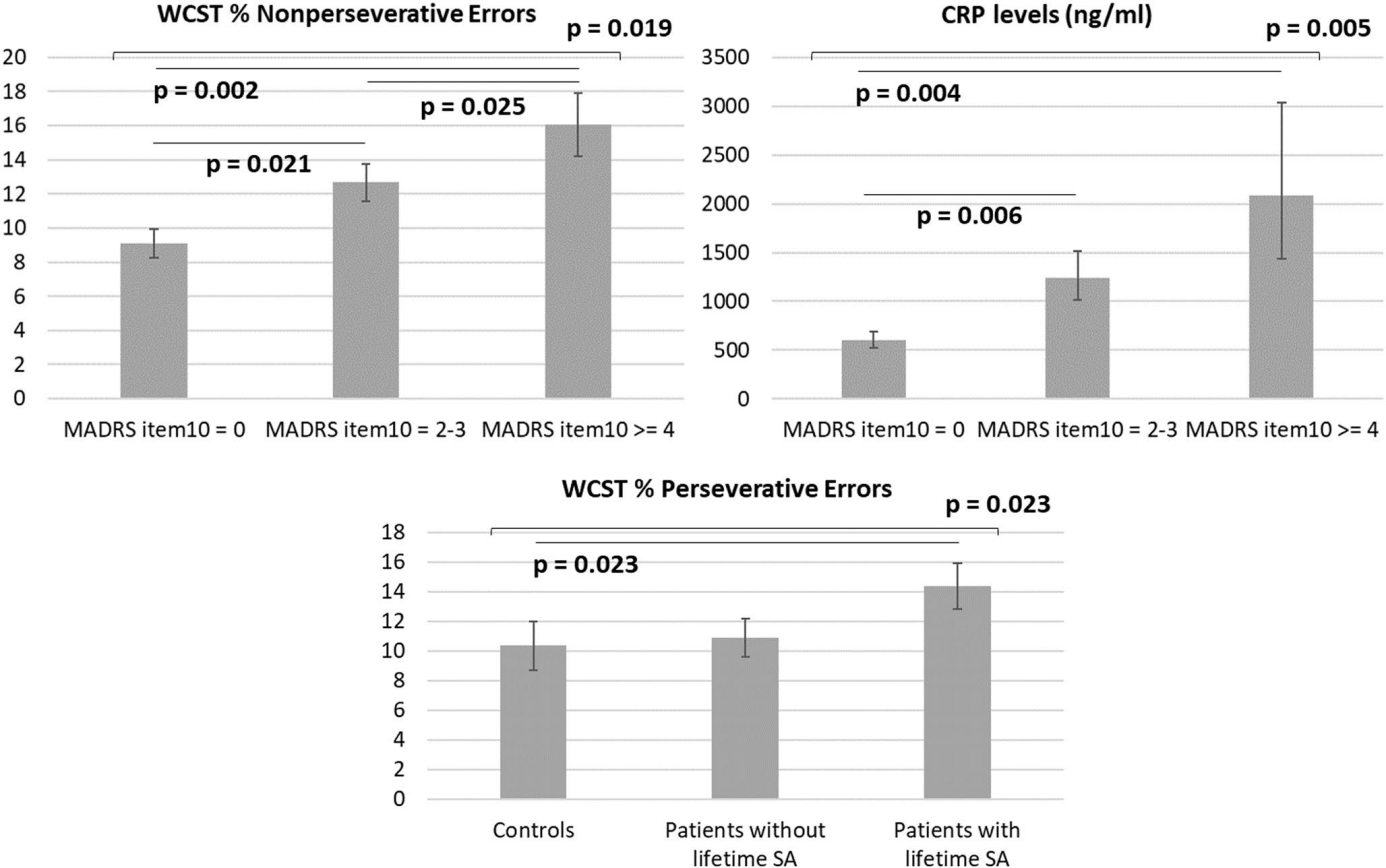
Estimated (means, standard errors) WCST results and CRP levels between groups using the generalized linear models. Note: adjusting for age, sex, BMI, education years, diagnoses, and non-suicidal depressive symptoms. MADRS: Montgomery–Åsberg Depression Rating Scale; WCST: Wisconsin Card Sorting Test; BMI: body mass index; CRP: C-reactive protein; SA: suicide attempts

The GLMs adjusted for demographic factors, diagnoses, and nonsuicidal depressive symptoms revealed the main effects of current suicidal symptom severity on the percentage of nonperseverative errors on the WCST (*p* = 0.019) and CRP levels (*p* = 0.005), as well as the main effect of lifetime suicidal attempts on the percentage of perseverative errors on the WCST (*p* = 0.023) (Table 3). Figure 1 showed the post-hoc analyses of the executive function and CRP levels between groups. The CRP levels and the percentage of nonperseverative errors on the WCST demonstrated a dose-dependent relationship correlated with the severity of current suicidal symptoms, categorized as none, mild, or strong. Specifically, compared with individuals without current suicidal symptoms, patients with strong current suicidal symptoms demonstrated the highest CRP levels (*p* = 0.004) and the highest percentage of nonperseverative errors on the WCST (*p* = 0.002), and patients with mild suicidal symptoms had the intermediate levels (*p* = 0.006 and 0.021, respectively) (Fig. 1). Finally, only patients with a lifetime history of attempted suicide had the increased percentage of perseverative errors on the WCST (*p* = 0.023) compared with the healthy young people (Fig. 1).

**Table 3:** Generalized linear models of the concurrent suicidal symptoms and history of attempted suicide with the WCST results and CRP levels

	Wald χ2	*p*-value	Wald χ2	*p*-value
	WCST: Number of categories completed		WCST: % Nonperseverative errors	
Concurrent MADRS suicide item severity (CS)	3.13	0.209	7.91	**0.019**
Lifetime history of attempted suicide (SA)	0.54	0.463	2.66	0.103
CS*SA	2.24	0.327	1.68	0.432
	WCST: % Perseverative errors		CRP	
Concurrent MADRS suicide item severity (CS)	2.32	0.314	10.57	**0.005**
Lifetime history of attempted suicide (SA)	5.13	**0.023**	0.00	0.997
CS*SA	0.41	0.815	3.49	0.175

Bold type indicates the statistical significance (*p* <  0.05)

Adjusting for age, sex, BMI, education years, diagnoses, and non-suicidal depressive symptoms

*MADRS* Montgomery–Åsberg depression rating scale, *s.d.* standard deviation, *WCST* Wisconsin card sorting test, *BMI* body mass index, *CRP* C-reactive protein

## Discussion

Our findings provide partial support for the hypothesis that patients with strong suicidal symptoms (MADRS item 10 ≥ 4) would exhibit the highest CRP levels and most impaired executive function compared with non-suicidal individuals, regardless of which major affective disorder they have. Patients who scored 2 or 3 on item 10 of the MADRS exhibited intermediate CRP levels and executive dysfunction. However, we only found an association between a lifetime history of suicide attempts and executive dysfunction and not between a lifetime history of suicide attempts and CRP levels.

As noted, increasing evidence has established an association between CRP levels and current suicidal ideation [7, 22]. A cross-sectional study of 150 adolescent inpatients with depression (91 with suicidal ideation and 59 without) reported that increased CRP levels indicate suicidal ideation, with a sensitivity of 0.593 and a specificity of 0.712 [22]. Another study, which included 58 young patients with depression and current suicidal ideation (mean age approximately 30 years) and 61 without, found a positive correlation between the intensity of current suicidal ideation and CRP levels [23]. Our study found a dose-dependent association between current suicidal symptom severity and CRP levels, suggesting that CRP levels may serve as a concurrent marker of suicidality. However, we found no association between CRP levels and suicide attempt history, which corresponds with Jha et al.’s results indicating a similar lack of association but conflicts with Melhem et al.’s findings that young people suicide attempt history was related to increased CRP levels [8, 24]. The inconsistency may be due to the lack of adjustment for current suicidal ideation severity, non-suicidal mood symptoms, and measurement timing in the previous studies’ investigation of an association between CRP levels and lifetime history of suicide attempts [8, 24]. In addition, discrepant findings may stem from heterogeneity in patient selection (e.g., different age ranges and medical comorbidity burdens), because age-related increases in systemic inflammation (including CRP) could modify the observed association between CRP levels and suicidal symptoms [25]. Replication in larger, age-stratified cohorts enriched for suicidal symptomatology would be warranted to confirm the robustness of these findings.

Two types of WCST errors, namely nonperseverative and perseverative errors, reflect distinct neurofunctioning related to suicidality [10, 11, 26]. A perseverative error suggests an inability to select a new perceptual category and difficulty shifting away from the formerly reinforced category, commonly reflecting prefrontal cortical function; a nonperseverative error occurs when an individual incorrectly sorts the cards without perseverating on the wrong response, typically serving as an indicator of non-frontal cortical function (i.e., parietal region) [26–29]. However, relative to that on perseverative errors, research on nonperseverative errors is lacking [26–29]. Shin et al. found that nonperseverative errors were associated with network disruption between the cognitive cerebellum and frontoparietal regions and impairment in the cerebro–ponto–cerebellar tract [30]. Our study found a distinct relationship between the severity of current suicidal symptoms and nonperseverative errors, whereas a lifetime history of suicide attempts was associated with perseverative errors. Schreiner et al. suggested that the suicidal thoughts and behaviors of adolescents with depression are associated with the resting-state functional dysconnectivity between the left posterior cingulate cortex and left cerebellum, as well as between the left precuneus and left primary motor and somatosensory cortices [31]. Gifuni et al. observed that a history of suicide attempts was associated with widespread gray matter volume reduction in the prefrontal region, such as the orbitofrontal cortex, the rostral middle frontal, superior frontal cortex, and the precentral gyrus, among adolescents with depression [32]. Neuropsychological studies also support our results, showing that people who have attempted suicide may have cognitive inflexibility and make more perseverative errors on the executive function test, thereby hindering their ability to devise alternative strategies in stressful situations [33, 34]. Furthermore, McGirr et al. discovered that the first-degree relatives of patients who died by suicide had significantly more perseverative errors compared with the healthy controls [35]. Taken alongside this evidence, our findings may suggest perseverative errors as a trait marker of suicidality and nonperseverative errors as a concurrent marker of suicidality among young people.

Our study has several limitations. First, we only assessed executive function using the WCST and CRP levels. Additional studies would be necessary to investigate the relationships between different aspects of cognitive function or other proinflammatory cytokines and suicidality among young people with major affective disorders. Second, to avoid worsening their emotional and suicidal symptoms, patients continued to take their psychotropic prescriptions during the executive function examination and CRP measurement, in line with ethical standards. While medication continuation may have influenced both neurocognitive performance and inflammatory indices, a fully “drug-free” design in individuals with acute suicidal symptoms can be ethically and practically difficult because treatment discontinuation may increase symptom exacerbation and suicide risk. We emphasized that future work should prioritize safe and ethically acceptable approaches to addressing medication-related confounding, such as recruiting medication-naïve participants and using carefully monitored and clinically indicated washouts only in low-risk contexts, to better account for treatment effects. Importantly, participant safety and timely clinical care remain paramount in suicide-related research.

To conclude, our study indicated that increased CRP levels and executive dysfunction were associated with suicidality among young people, including current suicidal symptoms and lifetime suicide attempt history. Our study noted dose-dependent relationships between the severity of current suicidal symptoms, higher CRP levels, and the percentage of nonperseverative errors on the WCST. A lifetime history of suicide attempts was associated with a higher percentage of perseverative errors on the WCST. Our results may support an integrated suicide risk assessment that combines symptom severity, inflammation, and executive function among youth with major affective disorders.
